# Supplementary material for: Broadly Applicable Control Approaches Improve Accuracy of ChIP-Seq Data
Source: Int J Mol Sci. 2023 May 25;24(11):9271. doi: 10.3390/ijms24119271 (PMC10252487; doi:10.3390/ijms24119271)
Supplement: Supplementary file 1 [file ijms-24-09271-s001.zip › Supplementary figures.pdf]

Figure S1

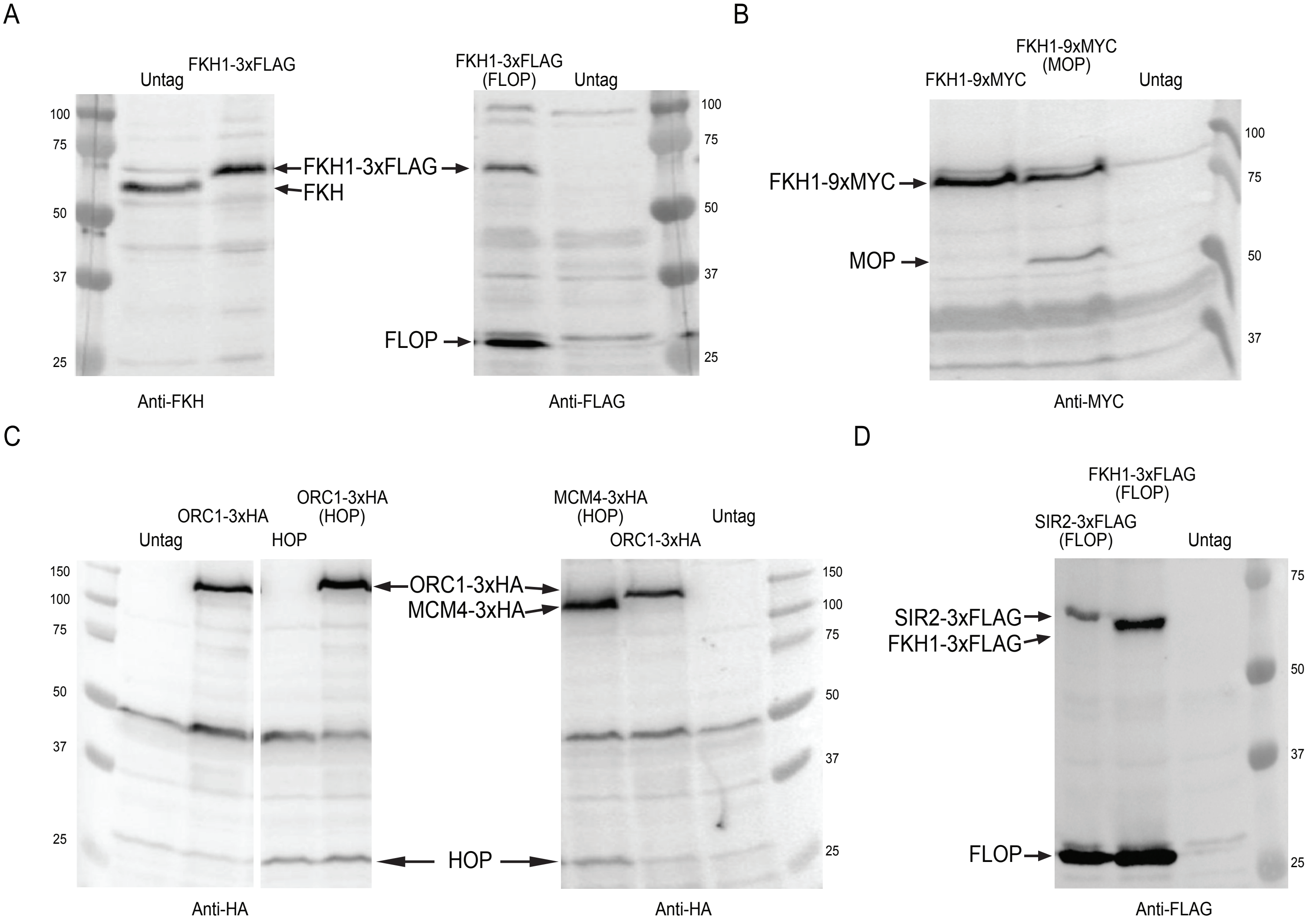

Supplement to Figure S1. Western blot analysis of epitope-tagged strains used throughout this study: “Untag” is SSy161 throughout,

(**A**) OAy1146 (FKH1-3xFLAG) and MPy55 (FKH1-3xFLAG FLOP).

(**B**) MPy166 (FKH1-9xMYC) and MPy108 (FKH1-9xMYC MOP).

(**C**) OAy503 (ORC1-3x-HA), MPy39 (HOP), MPy199 (ORC1-3xHA HOP), MPy184 (MCM4-3xHA HOP).

(**D**) YHy29 (SIR2-3xFLAG FLOP) MPy55 (FKH1-3xFLAG FLOP).

Figure S2

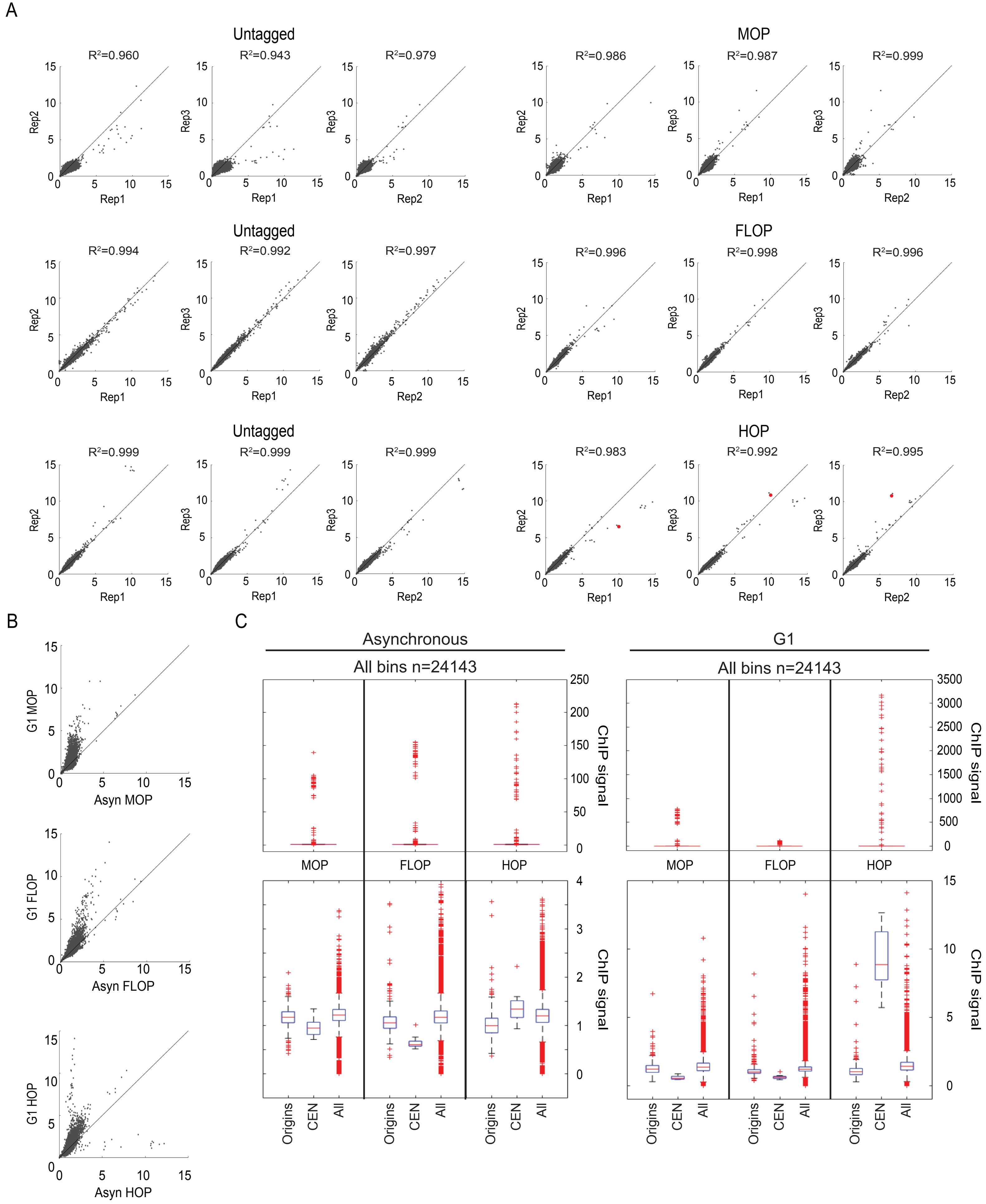

Supplement to Figure S2. (**A**) Two-dimensional scatter plots analyzing correlation of sample replicates. (**B**) Two-dimensional scatter plots of unsynchronized vs G1-synchronized MOP/FLOP/HOP data. (**C**) Boxplots of ChIP signals at replication origins and CENs.

Figure S3. (**A**) Two-dimensional scatter plots analyzing correlation of replicates. (**B**) Plots of ChIP signal across chromosomes III-L and IX with potential Fkh1 binding sites indicated as colored circles on one track and called peaks indicated on the lower track. (**C**) Heatmaps of averaged ChIP signal at snoRNAs.

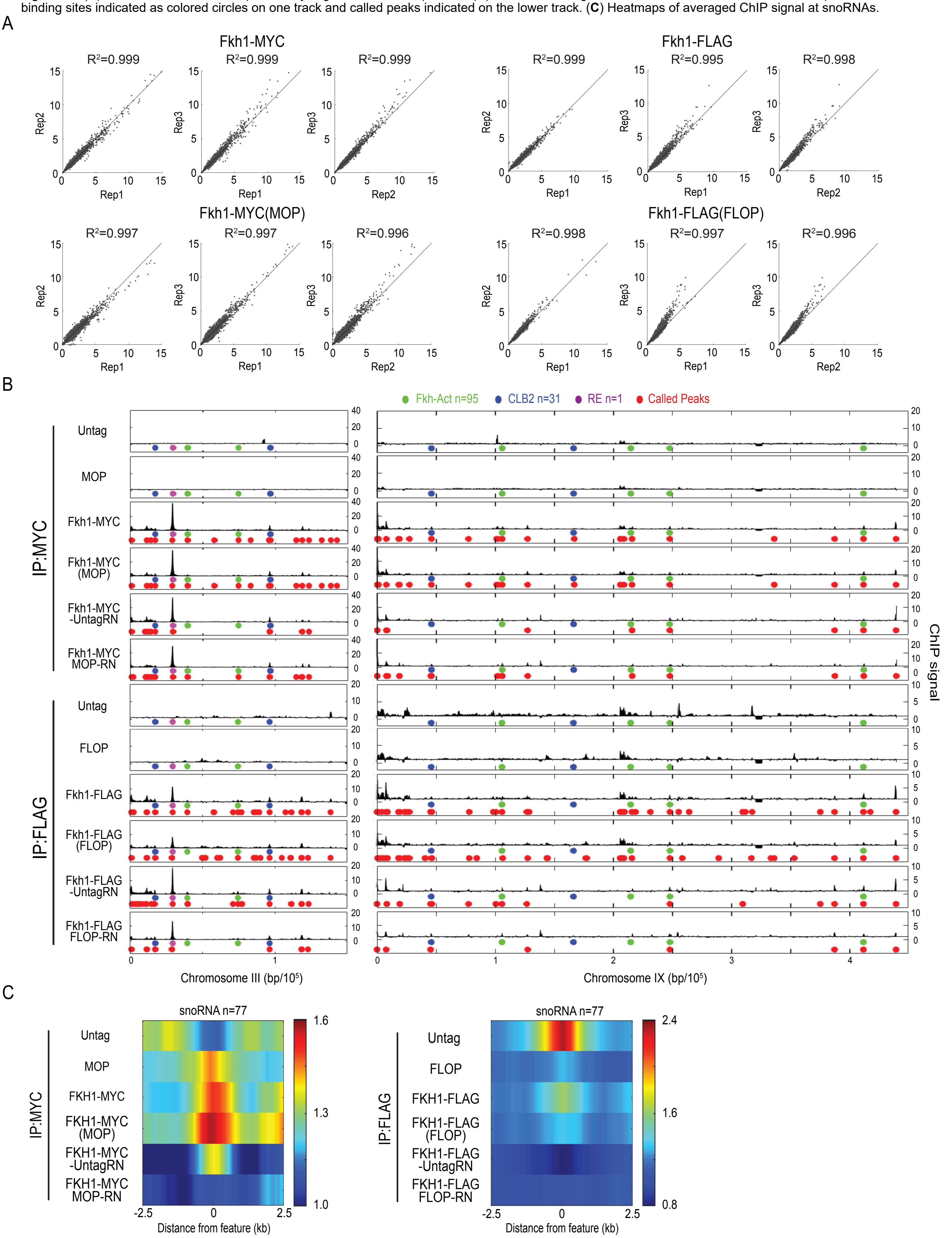

Figure S4

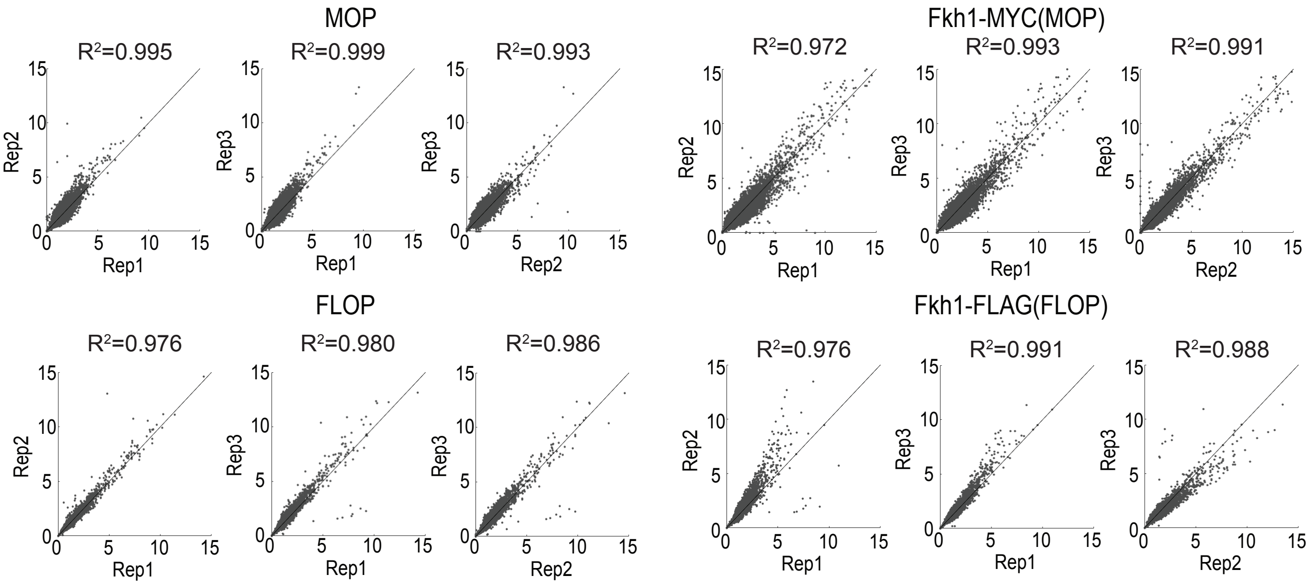

Supplement to Figure S4. Two-dimensional scatter plots analyzing correlation of replicates.

Figure S5

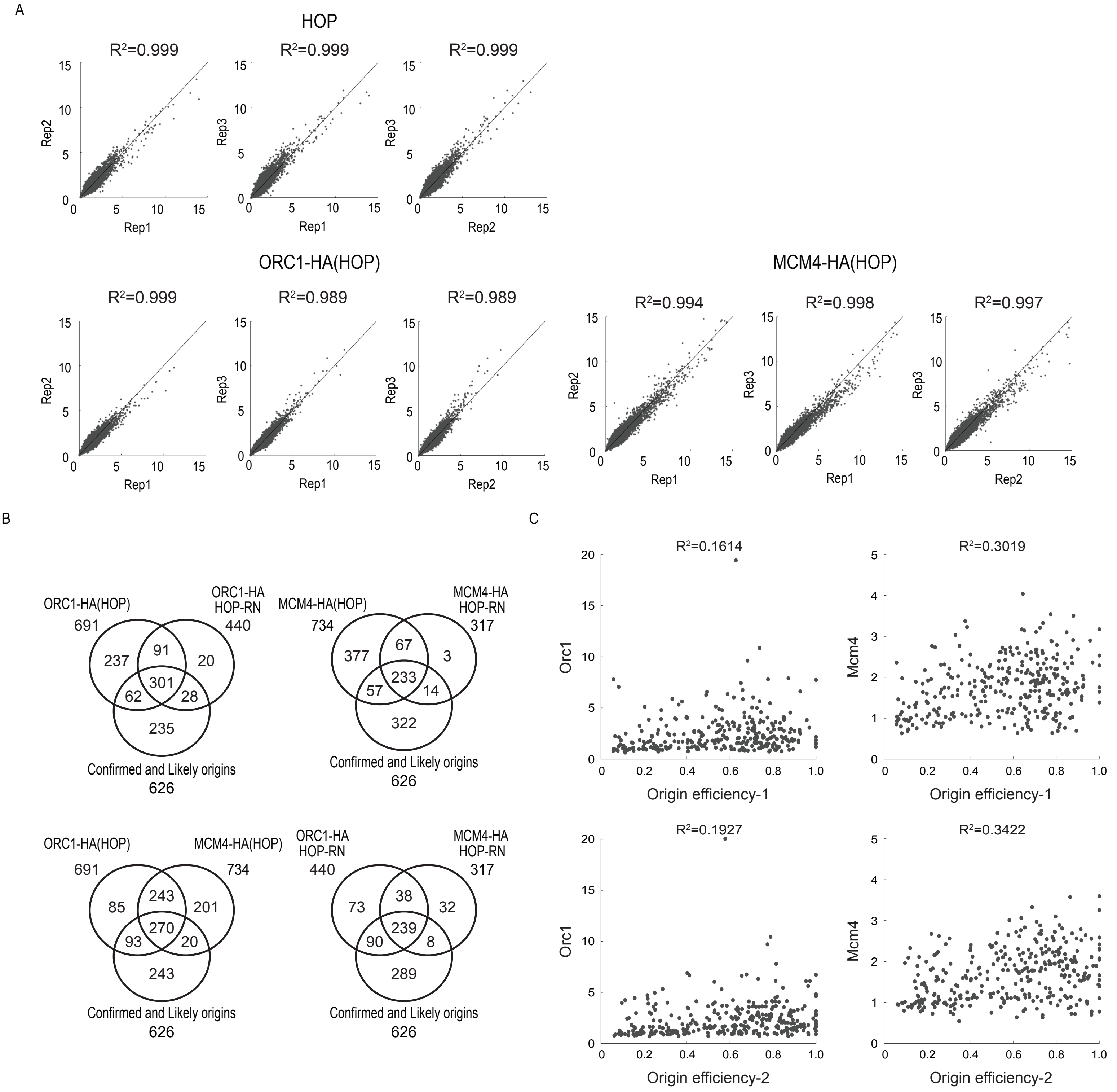

Supplement to Figure S5. (**A**) Two-dimensional scatter plots analyzing correlation of replicates. (**B**) Venn diagrams showing origin overlap of peaks called using RN and uncontrolled datasets against confirmed and likely origins. (**C**) Two-dimensional scatter plots comparing Orc1 and Mcm4 ChIP signals against origin efficiencies.

Figure S6

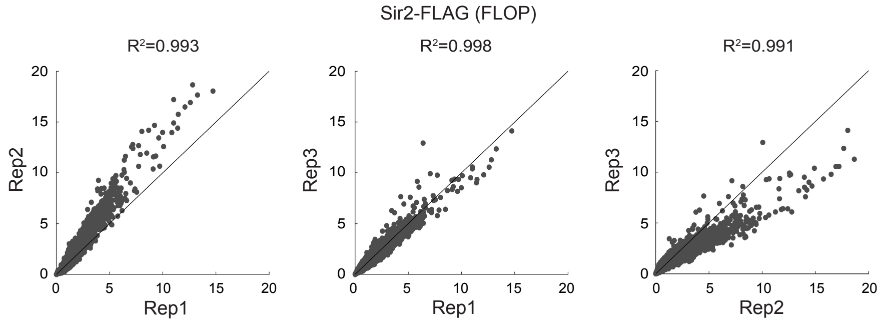

Supplement to Figure S6. Two-dimensional scatter plots analyzing correlation of replicates.

Figure S7

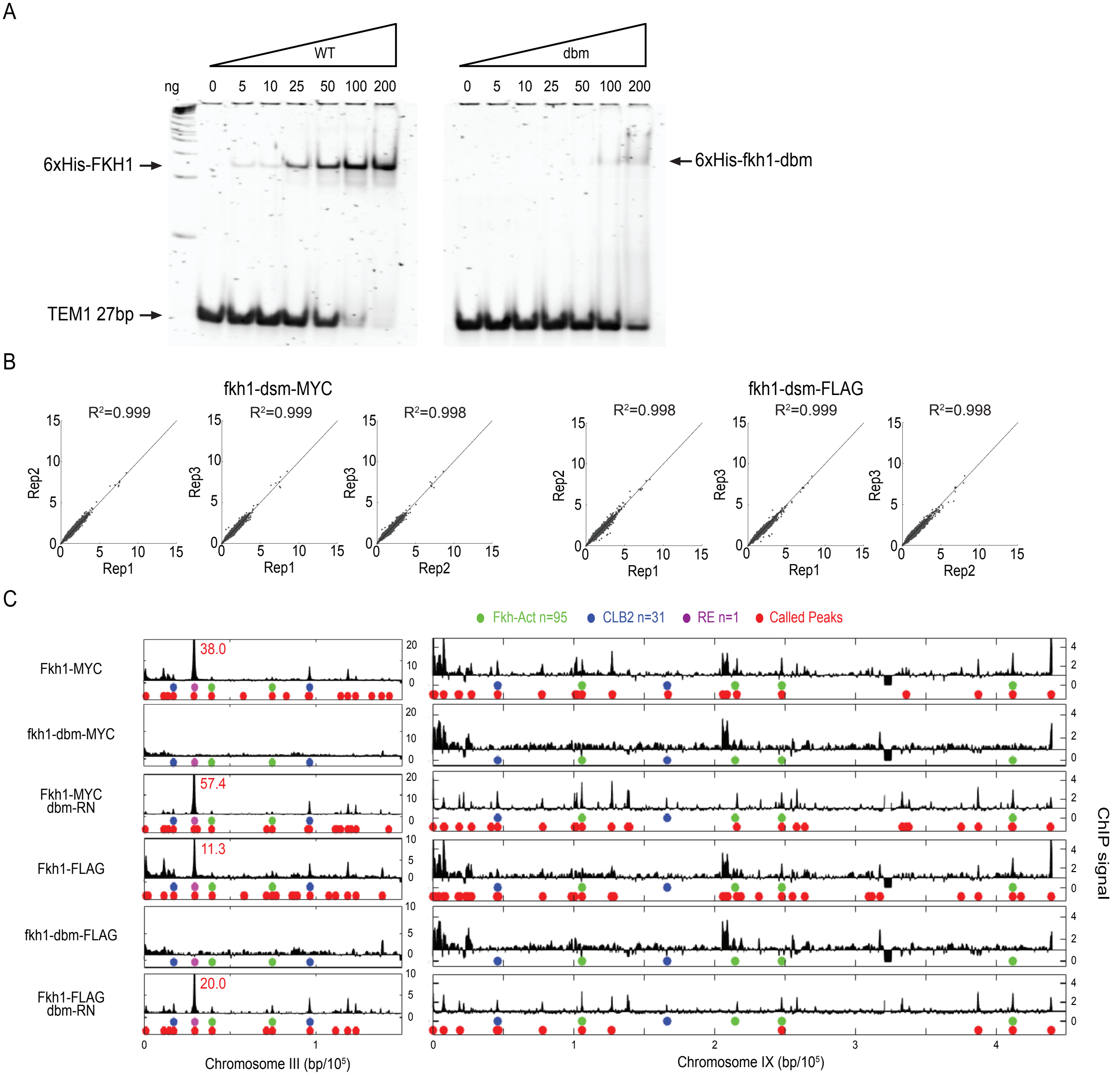

Supplement to Figure S7. (**A**) EMSA analysis of Fkh1-dbm versus Fkh1 binding to TEM1 DNA probe. (**B**) Two-dimensional scatter plots analyzing correlation of sample replicates. (**C**) Plots of ChIP signal across chromosomes III-L and IX with potential Fkh1 binding sites indicated as col-ored circles on one track and called peaks indicated on the lower track.
